# Preventing a global transition to thermoacoustic instability by targeting local dynamics

**DOI:** 10.1038/s41598-022-12951-6

**Published:** 2022-06-03

**Authors:** Nitin Babu George, Manikandan Raghunathan, Vishnu R Unni, R. I. Sujith, Jürgen Kurths, Elena Surovyatkina

**Affiliations:** 1 Potsdam Institute for Climate Impact Research, Potsdam, Germany; 2 Department of Physics, Humboldt University of Berlin, Berlin, Germany; 3 Department of Aerospace Engineering, Indian Institute of Technology, Madras, India; 4 Department of Mechanical and Aerospace Engineering, Indian Institute of Technology, Hyderabad, India; 5 Space Research Institute of Russian Academy of Sciences, Moscow, Russia

**Keywords:** Nonlinear phenomena, Phase transitions and critical phenomena, Aerospace engineering

## Abstract

The burning of fossil fuels to generate power produces harmful emissions. Lowering such emissions in gas turbine engines is possible by operating them at fuel-lean conditions. However, such strategies often fail because, under fuel-lean conditions, the combustors are prone to catastrophic high-amplitude oscillations known as thermoacoustic instability. We reveal that, as an operating parameter is varied in time, the transition to thermoacoustic instability is initiated at specific spatial regions before it is observed in larger regions of the combustor. We use two indicators to discover such inceptive regions: the growth of variance of fluctuations in spatially resolved heat release rate and its spatiotemporal evolution. In this study, we report experimental evidence of suppression of the global transition to thermoacoustic instability through targeted modification of local dynamics at the inceptive regions. We strategically arrange slots on the flame anchor, which, in turn, reduce the local heat release rate fluctuations at the inceptive regions and thus suppress the global transition to thermoacoustic instability. Our results open new perspectives for combustors that are more environmental-friendly.

## Introduction

Industrial gas turbine engines produce emissions such as NO_*x*_, CO and SO_*x*_ as by-products. A high-temperature combustion reaction is one of the key reasons for the formation of NO_*x*_^1^. Operating the gas-turbine engines at fuel-lean conditions by increasing the proportion of the oxidizer and thereby lowering the temperatures can reduce the harmful NO_*x*_ emissions. However, operating these engines at such fuel-lean conditions makes them prone to the phenomenon of thermoacoustic instability. Thermoacoustic instability is defined by self-sustained dangerous large-amplitude periodic oscillations of acoustic pressure and velocity. The phenomenon of thermoacoustic instability is a challenging problem faced in industrial-scale gas turbine engines, boilers, rockets^2–5^ as the associated vibration and thermal stress damage the structural integrity of the engines, navigation and control systems, thermal protection system and also results in the operational failure of engines^5–7^.

Thermoacoustic instability occurs because of flow–flame–acoustics interactions^2,8–10^. As the acoustic pressure and heat release rate fluctuations become in phase^11^, a positive feedback is established, which causes the growth of periodic temporal dynamics^2,3,5,6^ and periodic emergence of spatially organized patterns^7,12–14^. In order to operate the engines at environmental-friendly conditions, suppression of thermoacoustic instability is a necessity. However, in industrial-scale systems, the problem of thermoacoustic instability is particularly challenging due to highly turbulent flows, high operating pressures and power densities, multiple inlet systems as well as complex fuels^15^.

The suppression of thermoacoustic instability can be via two approaches: active and passive control strategies^15^. These control strategies intend to decouple the positive feedback between acoustic pressure and the heat release rate oscillations^16,17^. One example of a passive control strategy is to disrupt large-scale coherent vortical flow structures that emerge in the system by modifying the combustor geometry, through secondary air injections^18–20^. In some studies, secondary injections of fuel such as hydrogen are introduced into the combustion chamber to suppress thermoacoustic instability^21,22^. These injection strategies are often limited because they increase NOx emissions or alter the thermal power^22^. Notably, most of these strategies are based on the large-scale spatiotemporal dynamics such as coherent flow structures and strong temporal modes that appear during the occurrence of thermoacoustic instability.

Importantly, in the last decade, many studies have shown evidence of precursors of the upcoming transition, far before the appearance of large-scale spatiotemporal features^23–29^. In fact, this transition in turbulent thermoacoustic systems happens from the state of combustion noise (*S*_*C*_), via the state of intermittency (*S*_*IN*_) to the state of thermoacoustic instability (*S*_*TI*_), with a continuous increase in periodicity. Such a transition was identified experimentally and modeled in recent years^30–33^. During the transition, continuous change appears in spatial dynamics of the flow field and reaction field as well^34,35^. This continuous change in the spatiotemporal dynamics makes it difficult to pinpoint when (in time, or in parameter space) and where (spatial domain) the transition to thermoacoustic instability initiates. However, a prior study reports that such locations, which show early indications of the transition do exist^36^. Therefore, control measures could be effectively implemented well before the appearance of large-scale dynamics in the system. Multiple studies have shown early detection of an impending thermoacoustic instability by estimating measures based on the time series of pressure fluctuations^23,24^. In this study, we search for such corresponding spatial indicators of the onset, which could reveal certain locations that are vital for the suppression of the global transition to thermoacoustic instability.

In that context, we investigate the global transition from the state of combustion noise to the state of thermoacoustic instability as a phase transition, wherein the regime changes from disordered to ordered spatiotemporal dynamics^37,38^. Preventing undesirable phase transitions, in general, has been a tremendous scientific challenge with a long history in statistical physics, phase transition theory^39^, and critical fluctuations^40^. In nonlinear systems, the notion of instability implies that small fluctuations grow with time—a system that is “close” to being unstable is extremely sensitive to external perturbations^41^. At the phase transition from the fixed point to the periodic solution, the amplification of fluctuations of state variables appears as a universal property on the following grounds. At approaching the critical point, the initial stable state is losing its stability; the damping is reducing and tending to zero at the critical point^42^, leading to the growth of fluctuations^43^ . The amplification of fluctuations depends only on the type of dynamical instability involved (or the type of bifurcation), independent of the physics behind the governing differential equation^41^. Hence, critical fluctuations are precursors of upcoming phase transitions and nonlinear theory^44^ predicts the evolution of linear growth into a nonlinear saturation of the fluctuations near the critical point.

Amplification of fluctuations is also accompanied by another phenomenon—the increase in the correlation time of a state variable, *τ*_*c*_, that can be estimated near the critical threshold from the relation^45^

$$\begin{aligned} \tau _c |\lambda |\approx 1, \end{aligned}$$

where *λ* is a Lyapunov exponent indicating how close the system is to the critical threshold, $|\lambda |= 0$. Hence, the correlation time *τ*_*c*_ increases infinitely when $|\lambda |$ $\rightarrow$ 0. Nevertheless, according to nonlinear theory, the increase in *τ*_*c*_ also experiences saturation in the vicinity of the critical point^45^. After the critical point, coherence resonance^46^ also impacts the amplification of a correlation time by the noise pushing effect on the unstable system during the transition to the limit cycle oscillations regime. Thus, the application of regularities discovered in^44,45^ allows identifying critical transition and making predictions, for instance, in the examples listed below. For the first time, the questions where and when were answered in^47^ by identifying a transition to monsoon as a critical transition, discovering the tipping elements in the monsoon system, and proposing a long-term prediction of monsoon onset. Then the methodology in^47^ was applied and shaped for a thermoacoustic system, which revealed the seeds of phase transition—locations where thermoacoustic instability initiates^36^. In this study, we take a step forward to explore how to prevent the appearance of thermoacoustic instability by modifying the dynamics at such crucial locations—the seeds of the phase transition.

## Results

The components of the experimental setup, which is a turbulent combustor that is stabilized by a circular bluff body and its data acquisitions systems are given in the “Methods” section. In this study, we analyze acoustic pressure fluctuations $p^{\prime }$ obtained using a piezoelectric pressure transducer and the global heat release rate $\dot{Q}$ by employing a photomultiplier (PMT). Additionally, we analyze spatial chemiluminescence intensities that are representative of the local heat release rate ($\dot{q}({\mathbf{x}} ,t)$). The spatial chemiluminescence intensities are obtained by utilizing a high-speed camera. At first, we perform experiments utilizing the circular bluff body, referred to as the baseline (BL) case. We maintain a constant mass flow rate of fuel ($\dot{m}_{fuel}$) at 34 SLPM (standard litres per minute). We vary $\dot{m}_{air}$ (operating parameter) from 537 SLPM to 957 SLPM, such that the approximate global equivalence ratios *ϕ* that correspond to the upper and lower limit of air mass flow rates are 0.99 and 0.55 (Maximum uncertainty ± 0.2%). In this turbulent combustor, blowout occurs below *ϕ* = 0.3. Since the range of *ϕ* used in our study is between 0.99 and 0.55, it is reasonable to expect that blowout does not affect the spatiotemporal dynamics analyzed here.

### The spatio-temporal emergence of thermoacoustic instability

**Figure 1:**
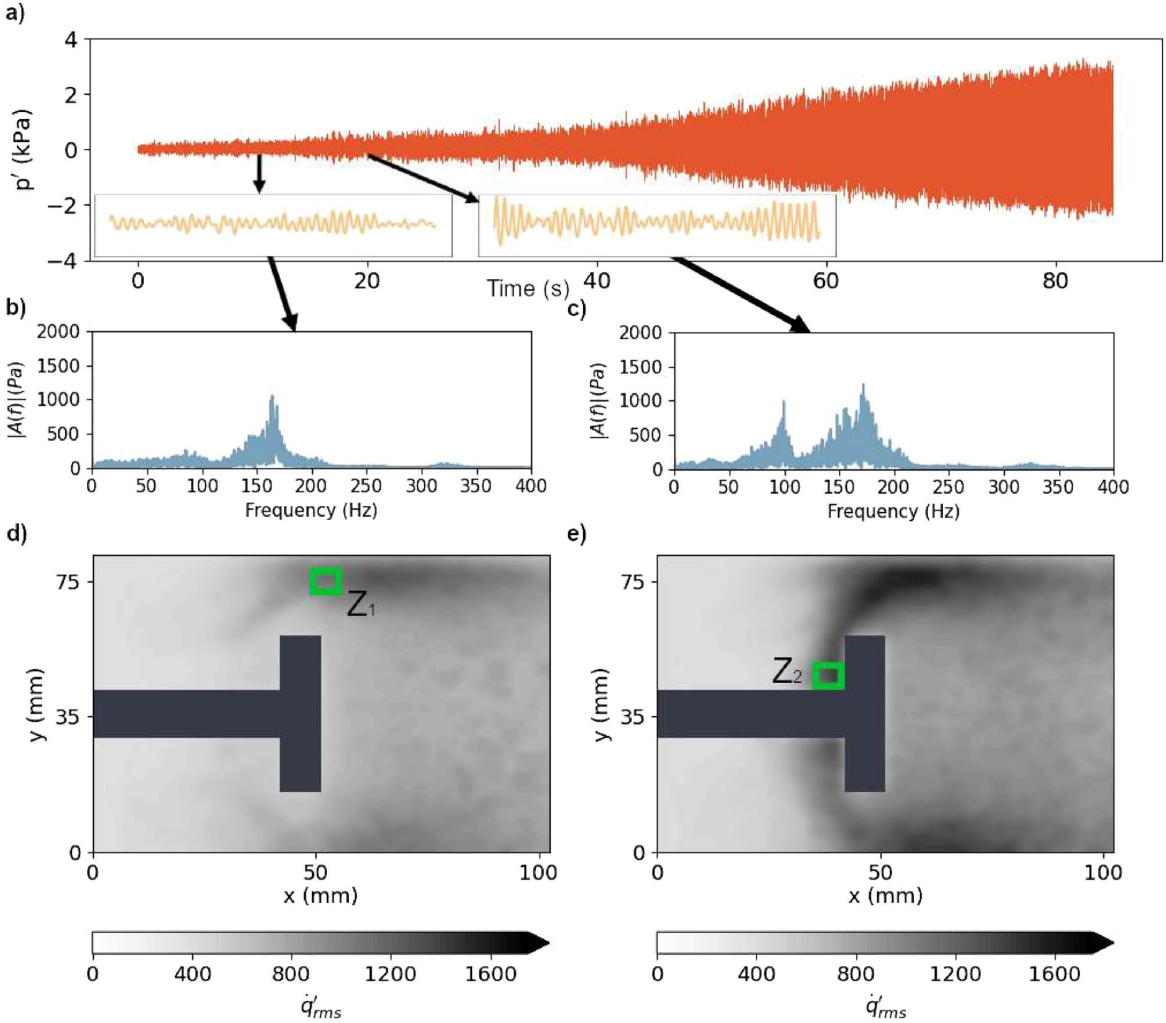
(**a**) Growth of pressure fluctuations from *S*_*CN*_ to *S*_*TI*_ for the BL bluff body. The insets indicate zoomed-in view of the pressure fluctuations at $t$ = 10 s and 20 s for 0.25 s. The corresponding amplitude spectra obtained from Fast Fourier Transform (FFT) of $p^{\prime }$ from *t* = 5–15 s (**b**) and from *t* = 15–25 s (**c**). As the control parameter is varied linearly in time, low-amplitude chaotic fluctuations change to high-amplitude periodic oscillations. At *t* = 0 s, $\dot{m}_{air}$ = 537 SLPM and at *t* = 80 s, $\dot{m}_{air}$ = 777 SLPM. The root mean square (*rms*) of local heat release rate fluctuations ($\dot{q}^{\prime }_{rms}$) at *t* = 10 s (**d**) and at *t* = 20 s (**e**). The bluff body and its shaft are marked with a black color. The locations Z_1_ and Z_2_ where high $\dot{q}^{\prime }_{rms}$ exist are illustrated by green squares.

Analysis of temporal dynamics of the state of the system unravels the growth of amplitude and periodicity in the oscillations enroute to thermoacoustic instability. Figure 1a shows that in the turbulent combustor, the thermoacoustic system transitions from a state characterized by low amplitude chaotic fluctuations (see Fig. S3 for tests for chaos) of acoustic pressure (*t* < 20 s) to a state characterized by high-amplitude periodic acoustic pressure oscillations (*t* > 60 s). This change in the dynamics occurs as $\dot{m}_{air}$ is increased (or in other words, as equivalence ratio is decreased). The plots of amplitude spectra obtained from the Fast Fourier Transform (FFT) of $p^{\prime }$ for 10 s, representative of the insets shown in Fig. 1a are displayed in Fig. 1b,c. The FFT of $p^{\prime }$ shown in Fig. 1b shows a distinct shallow band centered around a dominant frequency of 163 Hz. With increase in $\dot{m}_{air}$, this shallow band shifts slightly and becomes centered around 172 Hz and another shallow band centered around 100 Hz emerges (Fig. 1c).

The growth of pressure fluctuations in turbulent systems is gradual en route to thermoacoustic instability. However, various studies have shown that certain nonlinear measures corresponding to the state variables of the system show an early detection of thermoacoustic instability even before a significant growth in pressure oscillations^23,48,49^. We hypothesize that similar precursory measures of an impending onset of thermoacoustic instability could also be derived from the dynamics of a spatiotemporal variable measured at certain regions in space. Finding such regions may hold the key to identifying spatiotemporal precursors and unravelling passive control strategies that suppress the transition to thermoacoustic instability.

To examine the spatial dynamics near the transition, we analyze the spatially resolved local heat release rate fluctuations. Figure 1d,e show the spatial distribution of the root mean square of local heat release rate fluctuations $\dot{q}^{\prime }_{rms}({\mathbf{x}} ,t)$ at mass flow rates of air $\dot{m}_{air}$ = 550 SLPM (Fig. 1d) and 650 SLPM (Fig. 1e) respectively for the BL case. The *rms* is calculated by using one second windows at $\dot{m}_{air}$ = 550 SLPM (*t* = 4 s) and 650 SLPM (*t* = 38 s). Notably, at $\dot{m}_{air}$ = 550 SLPM, we observe high $\dot{q}^{\prime }_{rms}$ above the bluff body and also at the corner between the bluff body and the shaft. We mark two zones, Z_1_ and Z_2_ with green squares that represent regions of high $\dot{q}^{\prime }_{rms}$. Z_1_ is above the bluff body (Fig. 1d), while Z_2_ is at the corner between the bluff body and the shaft (Fig. 1e). The images of instantaneous heat release rate (Fig. S1) near *t* = 10 s and 20 s support the findings that fluctuations of the heat release rate at Z_2_ grow as the mass flow rate of air is increased. Thus, the strength of local heat release rate fluctuations is neither uniform nor growing evenly at all locations within the turbulent combustor.

**Figure 2:**
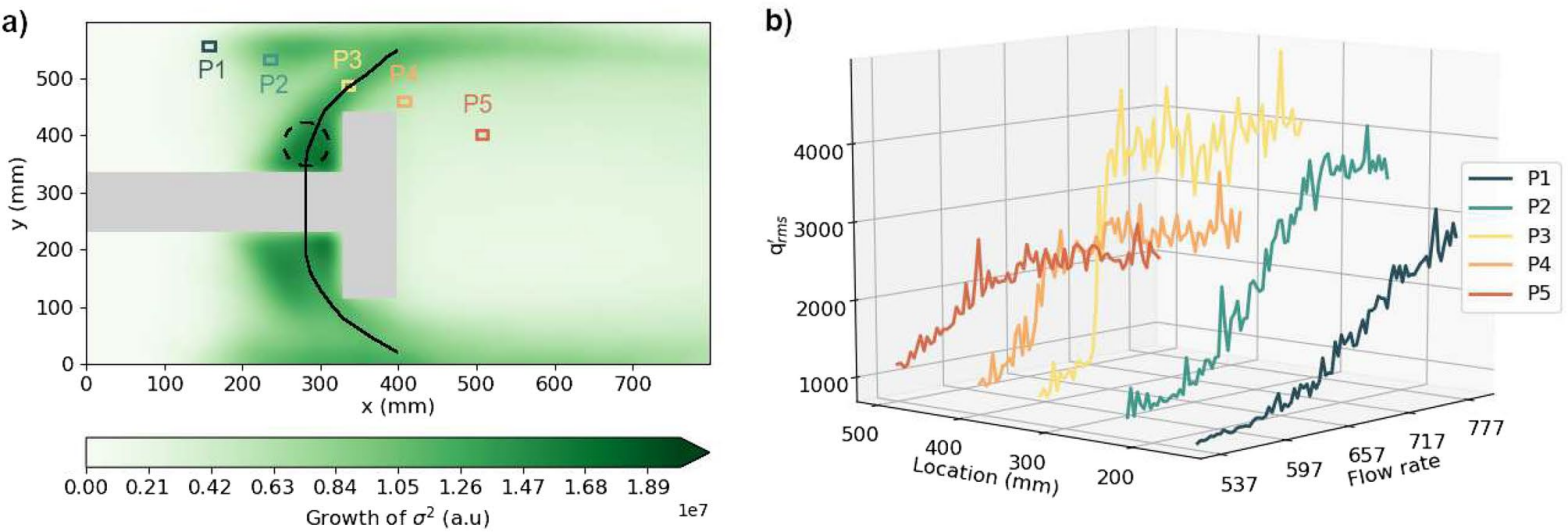
(**a**) The growth of *σ*^2^ (variance of fluctuations) of spatially resolved $\dot{q}^{\prime }$, calculated as the difference of *σ*^2^ at $\dot{m}_{air}$ = 600 SLPM and $\dot{m}_{air}$ = 650 SLPM for the BL bluff body. The growth of *σ*^2^ is high at certain regions, marked a C-shaped black curve. Colored squares indicate the locations that we utilize to plot the local $\dot{q}^{\prime }_{rms}$ in (**b**). The dashed black circle represents the region Z_2_. (**b**) The local $\dot{q}^{\prime }_{rms}$ at P1, P2, P3, P4 and P5 at different flow rates. As the control parameter is varied, a spatiotemporal pattern can be observed in the transition from S_*CN*_ to S_*TI*_ with a gradual transition at P1, an abrupt transition at P3 and a disappearance of the abruptness of the transition at P5.

Z_1_ is a location of bursts of high heat release rate resulting from the occasional impingement of large-scale coherent flow structures^34^. On the other hand, the corner between the shaft and the bluff body where Z_2_ is located provides a low-velocity region vital for flame stabilization^4^. In Fig. 1e we observe that at both $\dot{m}_{air}$ = 650 SLPM, high $\dot{q}^{\prime }_{rms}$ expands to a larger area downstream and upstream of the bluff body. It is important to note that at $\dot{m}_{air}$ = 550 SLPM and 650 SLPM, the thermoacoustic system is well before the appearance of large-scale patterns and at these conditions, the flame fluctuations are strongest near the bluff body. Due to the combustor’s geometry with respect to the shaft, we observe axial symmetry in the distribution of $\dot{q}^{\prime }_{rms}$ (Fig. 1d,e).

Our analysis of the phase transition to thermoacoustic instability reveals a predisposition for local flame fluctuations to grow at certain regions. We calculate the growth of fluctuations^44^ in the local heat release rate prior to the phase transition by estimating the difference in the variance of fluctuations *σ*^2^ of $\dot{q}^{\prime }({\mathbf{x}} ,t)$ between $\dot{m}_{air}$ = 550 SLPM and 650 SLPM (Fig. 2a). The calculation of *σ*^2^ for $\dot{q}^{\prime }({\mathbf{x}} ,t)$ is described in “Methods”. We observe that, around the bluff body, there is a C-shaped zone that connects Z_1_ and Z_2_, which exhibits a high growth of *σ*^2^ of $\dot{q}^{\prime }({\mathbf{x}} ,t)$. Meanwhile, the wake and the corner recirculation zones exhibit a low growth in *σ*^2^. Notably, far before the appearance of large-scale patterns in the spatiotemporal dynamics (i.e. during thermoacoustic instability), the highest growth in *σ*^2^ is observed at Z_2_, marked by a dashed black circle. To realize the importance of Z_2_ with respect to the phase transition, in Fig. 2b, we plot the local $\dot{q}^{\prime }_{rms}$ at regions P1 to P5, indicated in Fig. 2a.

Interestingly, we also observe a spatiotemporal expansion of the local transition in the dynamics of state variables ($\dot{q}^{\prime }_{rms}({\mathbf{x}} ,t)$), eventually resulting in the global phase transition to thermoacoustic instability as explained below. At P1, the transition from low $\dot{q}^{\prime }_{rms}$ to high $\dot{q}^{\prime }_{rms}$ occurs gradually (Fig. 2b) as $\dot{m}_{air}$ is increased. At P2, the transition occurs more abruptly compared to P1. Further, at higher $\dot{m}_{air}$, $\dot{q}^{\prime }_{rms}$ at P2 is higher than at P1. At P3, which is within the C-shaped zone, the transition is the most abrupt, when compared to P2 and P1. Further, the abrupt increase in $\dot{q}^{\prime }_{rms}$ occurs very early, near $\dot{m}_{air}$ = 650 SLPM. As we move further down the combustor, at P4, the transition occurs early, but the abruptness of the transition is lower compared to P3. Finally, at P5, which represents the dynamics in the wake, the abrupt transition ceases to exist and further increase in $\dot{q}^{\prime }_{rms}$ does not occur beyond $\dot{m}_{air}$ = 650 SLPM. Thus, a change in the criticality of the transition in the spatial dynamics occurs within the system itself as we move from upstream of the bluff body to downstream of the bluff body. While we observe a change in the criticality in the transition as we move from one location to another, previous studies show that a similar change in criticality also occurs when some parameter of the system is changed^50^. In our study, given that the transition in spatiotemporal dynamics initiates and appears to permeate from this C-shaped region, notably at Z_2_ (Fig. 2a), we investigate the transition when this C-shaped pattern is disrupted.

### Modification of the bluff-body design

**Figure 3:**
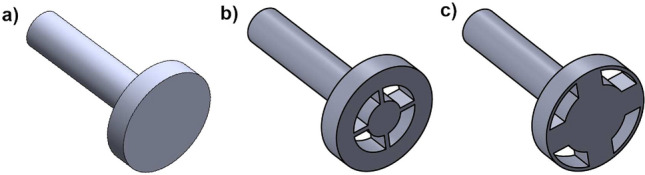
Schematic of the bluff bodies. (**a**) Baseline bluff body without slots. (**b**) Bluff body with slots near the center—inner slot bluff body (IS). (**c**) Bluff body with slots near the edge of the circle—outer slot bluff body (OS). Both slotted bluff bodies have the same area of slots.

Before discussing the strategy to suppress the phase transition, it is vital to see the relation between the dynamics at flame stabilization regions and at Z_2_. In our system, the flame has multiple regions of stabilization (see Supplementary Fig. S2), the recirculation zone next to the dump plane and the stagnation point upstream of the bluff body^51^. The region Z_2_ where we observe critical phenomena is near the stagnation point for the bluff-body seen in Fig. 3a. The control strategy that we employ is to reduce the heat release rate fluctuations near this stagnation point. In particular, we insert slots on the bluff body (The area of the slots is 36% of the cross-sectional area of the bluff body) to make it porous and create additional paths for the fluid to flow across the bluff body. In this manner, we distribute the heat release rate into a larger area so that the perturbations in heat release rate stemming from flow fluctuations are not necessarily localized to a small region. Even in the presence of slots, the bulk flow from the inlet stream is still around the bluff body as the area of the slots is only 7% of the cross flow area in the combustor.

Utilizing new designs of the bluff body, we modify the flow patterns with the intention of redistributing the heat release rate to a larger area and thereby reduce such fluctuations at Z_2_ to suppress the global transition to thermoacoustic instability. These designs are shown in Fig. 3b,c. In the first design, the slots are centered around the shaft (Fig. 3b). In this design, the hollow space corresponds to an area of 450 mm^2^. This hollow space is made between *r* = 8–15 mm. We refer to this design as inner slot bluff body (IS). In the second design, we introduce these slots near the edge of the bluff body, between *r* = 15 mm and 22 mm (Fig. 3c). We keep the same area of the hollow space for both the designs. We refer to this design as the outer slot bluff body (OS).

### Suppression of the local growth of *σ*^2^ at the C-shaped region and the global transition to thermoacoustic instability

Experiments with the modified designs of the bluff body show a redistribution of the local heat release rate and is discussed below. We perform a new set of experiments with the BL, IS and OS bluff bodies by varying the mass flow rates of air from 537 SLPM to 957 SLPM and keeping the mass flow rate of fuel ($\dot{m}_{fuel}$) fixed at 34 SLPM. We acquire the same set of measurements as earlier with the same instrumentation and sampling frequencies. First, we analyze the growth of *σ*^2^ of $\dot{q}^{\prime }({\mathbf{x}} ,t)$, obtained from the high-speed camera that records CH^*^ chemiluminescence. We calculate the growth of *σ*^2^ between $\dot{m}_{air}$ = 550 SLPM and 650 SLPM by the difference in *σ*^2^ at these two mass flow rates.

**Figure 4:**
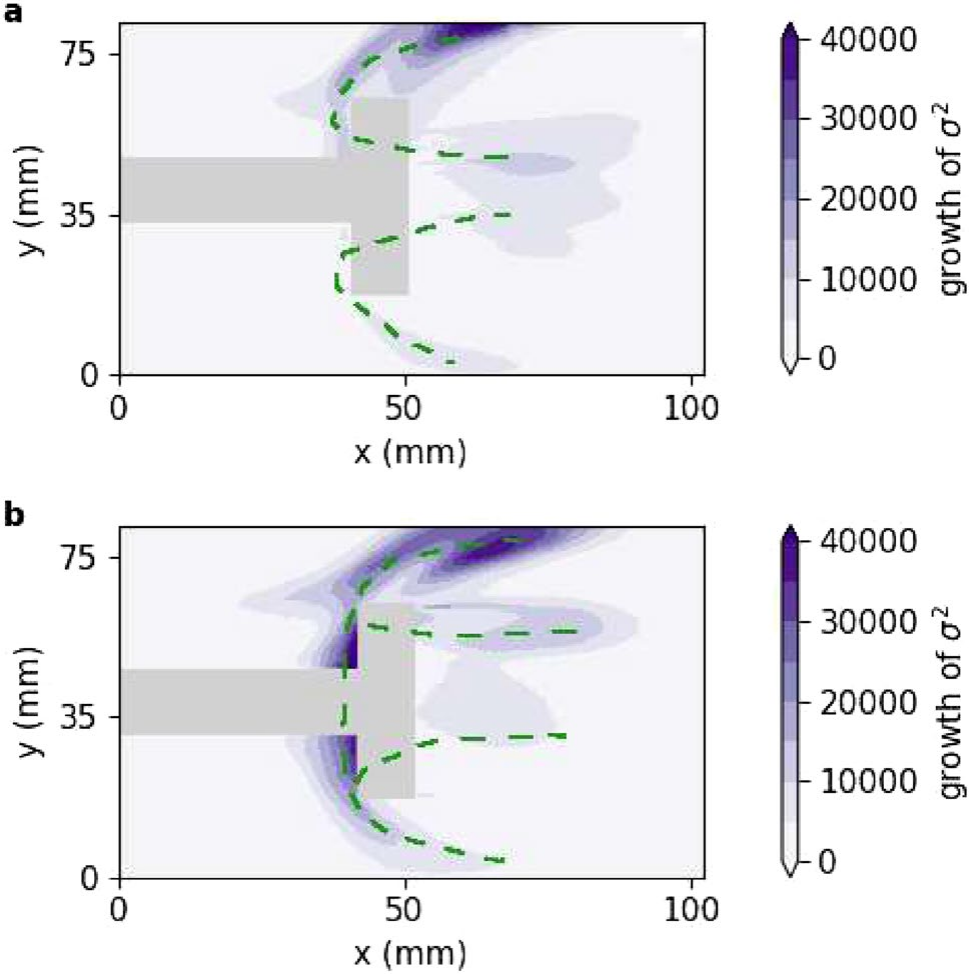
Growth of *σ*^2^ of local heat release rate fluctuations between two values of the control parameter $\dot{m}_{air}$ = 550 SLPM and 650 SLPM for experiments with (**a**) IS and (**b**) OS. For IS, growth of *σ*^2^ is negligible at Z_2_ while for OS, there is growth of *σ*^2^ at Z_2_. Regions of high growth of *σ*^2^ are highlighted using dashed green curves.

We redistribute the heat release rate fluctuations by modifying the flow pattern with the IS bluff body. This redistribution results in stifling the growth of *σ*^2^ at Z_2_ as $\dot{m}_{air}$ is increased from 550 SLPM to 650 SLPM. We observe a growth of *σ*^2^ only near Z_1_, but is shifted downstream (see Fig. 4a). As *σ*^2^ does not grow at Z_2_, the C-shaped structure breaks down and disconnected structures are formed, as shown in Fig. 4a. Further, there is some growth of *σ*^2^ in the wake, as a result of the slotted bluff body, although not as high as seen within the C-shaped structure for the BL case. In fact, high growth of *σ*^2^ in the wake occurs downstream of the IS as they also become locations for flame stabilization. However, we observe that *σ*^2^ does not keep growing at the wake to provide any new pathways to thermoacoustic instability.

Alternatively, for experiments with the OS bluff body, we find that the growth of *σ*^2^ is not suppressed at Z_2_. Figure 4b shows a high growth of *σ*^2^ both at Z_1_ and Z_2_. Additionally, we observe a growth of *σ*^2^ in the wake, similar to the results obtained with the IS bluff body.

**Figure 5:**
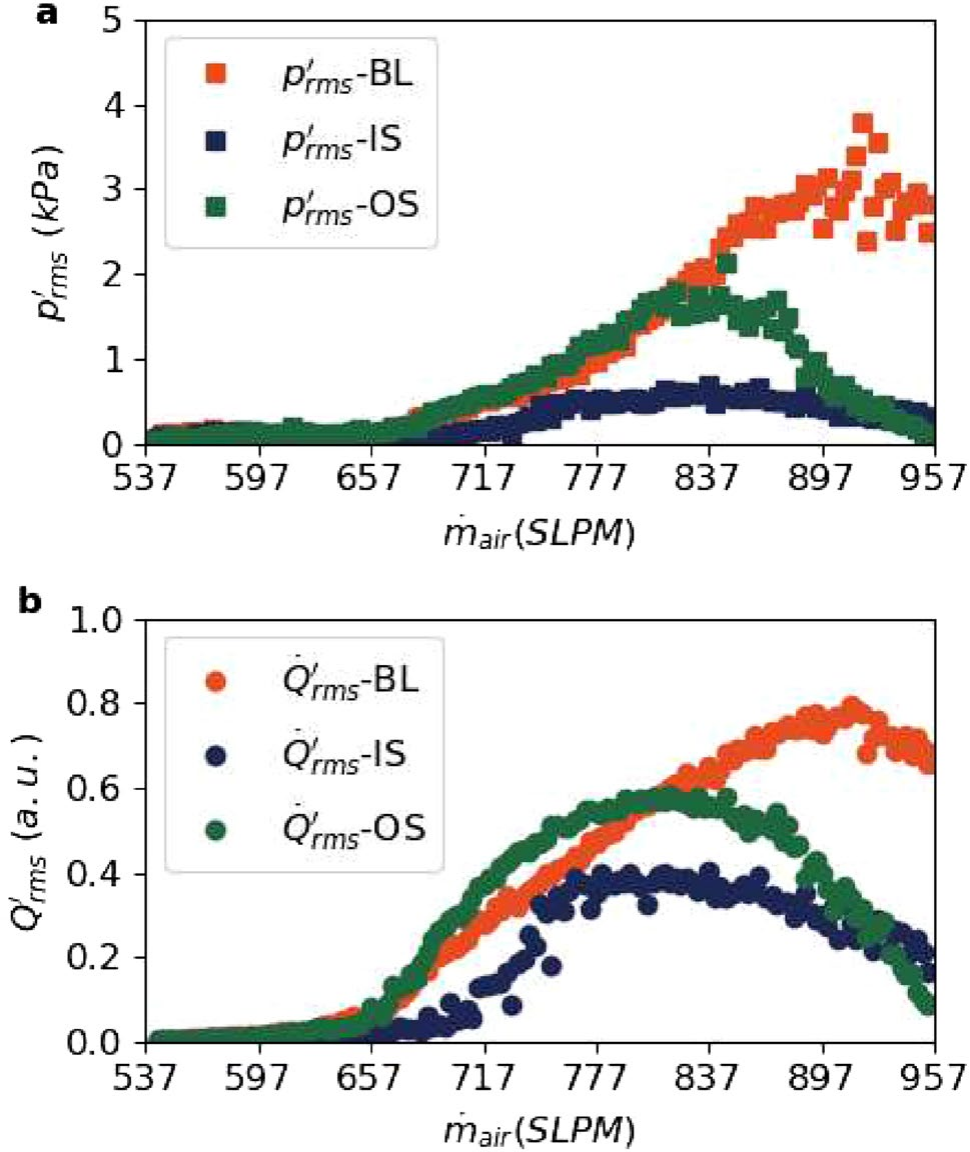
Time series of *rms* of the state variables that represent the global state of the system: $p^{\prime }$ and global heat release rate fluctuations: $\dot{Q}^{\prime }$ that is obtained from PMT for experiments with baseline design of bluff body, IS and OS. (**a**) $p^{\prime }_{rms}$ for BL, IS and OS bluff bodies and (**b**) $\dot{Q}^{\prime }_{rms}$ for BL, IS and OS bluff bodies. The global transition to thermoacoustic instability is suppressed for IS, while the suppression is not as effective for OS.

Now, we compare the measures of $p^{\prime }_{rms}$ and $\dot{Q}^{\prime }_{rms}$ in Fig. 5a,b respectively to show the suppression of the global transition to thermoacoustic instability. For BL, the highest value of $p^{\prime }_{rms}$ for BL is close to 4 kPa. Similarly, we observe a growth in $\dot{Q}^{\prime }_{rms}$ from $\dot{m}_{air}$ = 600 SLPM for the BL bluff body.

On the other hand, $p^{\prime }_{rms}$ of IS exhibits a minimal growth with a maximum value 0.55 kPa. In fact, the highest value of $p^{\prime }_{rms}$ for IS is 88% lower than the highest value of $p^{\prime }_{rms}$ for BL. However, the maximum value of $p^{\prime }_{rms}$ for IS is about 5.5 times the lowest value of $p^{\prime }_{rms}$ observed during combustion noise for BL. $\dot{Q}^{\prime }_{rms}$ increases only to about 50% of the highest values with BL (Fig. 5b).

For the OS bluff body, there is a high growth in $p^{\prime }_{rms}$ between 60 s and 100 s with the highest value around 2 kPa, which achieves a suppression of only 50% (Fig. 5a). The maximum value of $p^{\prime }_{rms}$ for OS is about 20 times the lowest value of $p^{\prime }_{rms}$ observed during combustion noise for BL. Further, $\dot{Q}^{\prime }_{rms}$ of OS experiences high growth and in fact increases earlier compared to BL (Fig. 5b).

Indeed, in the experiment with IS bluff body, stifling the growth of *σ*^2^ at Z_2_ inevitably results in the suppression of the transition to thermoacoustic instability. On the other hand, for experiments with BL and OS bluff bodies, there is a growth of *σ*^2^ at Z_2_ and the onset of high-amplitude oscillations occur at low equivalence ratios. The results using the OS bluff body demonstrates that a mere introduction of the slots that creates additional paths around the bluff body is insufficient to suppress the onset of thermoacoustic instability. We find similar results when we change the operating conditions of mass flow rate of fuel and rate of change of mass flow rate of air (see Supplementary Fig. S4).

**Figure 6:**
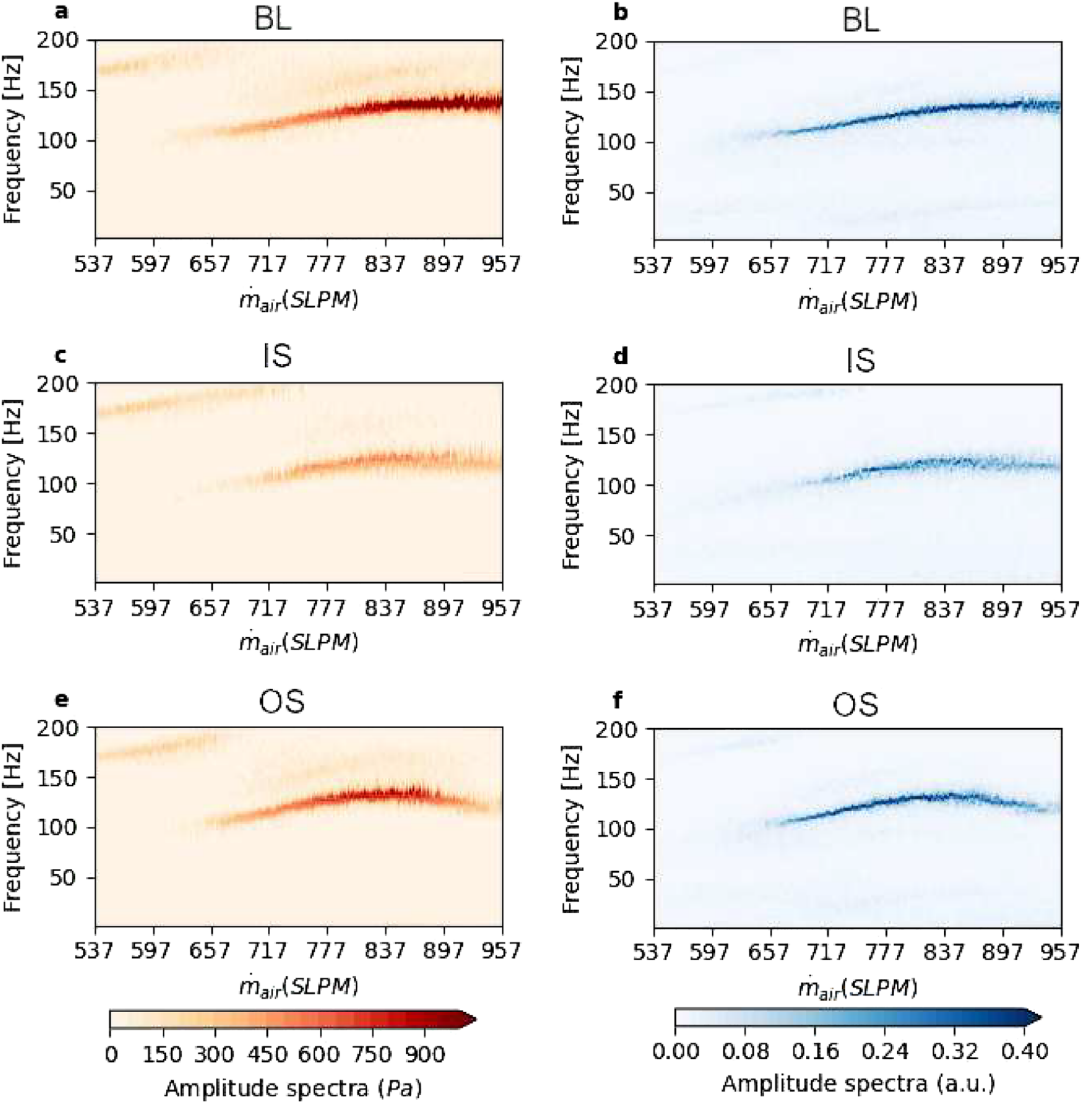
Evolution of the amplitude spectra obtained from short-time Fourier transform of the $p^{\prime }$ (**a**,**c**,**e**) and $\dot{Q}^{\prime }$ (**b**,**d**,**f**) time series for BL bluff body (**a**,**b**), IS bluff body (**c**,**d**) and OS bluff body (**e**,**f**). With the introduction of the slots, additional dominant frequencies do not emerge in comparison to BL.

Figure 6 shows the moving short-time Fourier transform (STFT) of $p^{\prime }$ and $\dot{Q}^{\prime }$ for the three different bluff bodies. For the BL bluff body, at $\dot{m}_{air}$ = 550 SLPM, we observe a high amplitude of pressure oscillations near a frequency of 175 Hz. Beyond 657 SLPM (*ϕ* = 0.81), we observe dominant frequencies that emerge near 100 Hz for both $p^{\prime }$ (Fig. 6a) and $\dot{Q}^{\prime }$ (Fig. 6b) for the BL. With an increase in $\dot{m}_{air}$, the dominant frequency increases and reaches an asymptotic value near 134 Hz, which is near the fundamental frequency of a quarter wave mode in the combustion duct. This increase in the dominant frequency occurs due to the periodic emergence of large-scale flow structures during phase synchronization and corresponds to the increase in $\dot{m}_{air}$^52^. Once generalized synchronization occurs, locking to the acoustic frequency happens and there is no further increase in the dominant frequency even though $\dot{m}_{air}$ increases^52^. Prior to $\dot{m}_{air}$ = 657 SLPM, we observe shallow bands centered around 164 Hz for $p^{\prime }$ and for $\dot{Q}^{\prime }$, centered around 25 Hz. This low frequency at 25 Hz corresponds to hydrodynamic fluctuations. Beyond 657 SLPM, there is a switch in the dominant frequencies to a single dominant frequency (Fig. 6a,b).

For the IS bluff body (Fig. 6c,d), at $\dot{m}_{air}$ = 550 SLPM, frequencies near 175 Hz are still dominant for $p^{\prime }$. Beyond $\dot{m}_{air}$ = 650 SLPM, a dominant mode emerges near 100 Hz, but not as strong as seen with the BL bluff body.

In the case of the OS bluff body (Fig. 6e,f), the mode near 130 Hz is strong, similar to the STFT of BL bluff body. These STFT plots give evidence that the suppression strategy devised by introducing passages in the bluff body with the IS and OS bluff bodies do not introduce other dominant frequencies but indeed suppresses the phase transition to periodic oscillations, notably for the IS bluff body.

## Discussion

Many undesirable phase transitions in the real world are of great interest to a broad scientific community to find suppression strategies^53–58^. Examples of such cases range from the onset of epilepsy^59,60^ to the occurrence of landslides^61,62^ and current instabilities in semiconductors^63,64^ to the failure of materials^65–67^ to name a few. However, it is a great challenge in each scientific field and multiple strategies may be needed. For example, for the prevention of the onset of epilepsy through deep brain stimulation, several targets on the brain are chosen dependent on the type of seizures such as partial seizures, generalized epilepsy or mesial temporal lobe epilepsy^59,60^.

Analogous situations appear in thermoacoustic systems. Thermoacoustic instability arises in different forms of instability such as annular modes, radial modes, axial modes in the combustion chamber. In our lab-scale turbulent thermoacoustic system, axial instabilities that emerge in the bluff-body stabilized flame are suppressed by targeted control strategies at a location that exhibits the earliest and most abrupt transition in flame fluctuations (Z_2_).

Any application of such strategies introduces additional dynamics in the system which may have stabilizing or destabilizing effects^68,69^. A successful mitigation of the transition depends on whether the stabilizing effects dominate over the destabilizing effects. In this paper, we have demonstrated that a certain design of the bluff body (IS) indeed suppresses the onset of thermoacoustic instability. However, this is not a one-size-fits-all strategy. Thus, formulating the correct strategy is an optimization problem that should be addressed in such complex systems. While in the laboratory setup, we were able to arrive at an optimal design for the flame holder, in real systems, where the topology of the combustor and flame holder is complicated, the topological optimization to suppress such transitions needs further research, which will be taken up as a future study. Furthermore, instead of using slots or perforations on the bluff body, secondary air/fuel injections may also be a useful method to target the local growth of fluctuations at the most critical regions.

In essence, we revealed that specific regions of a certain spatiotemporal system may exhibit precursory transitions in the dynamics of state variables that mirror the impending global phase transition of the system. The control of thermoacoustic instability would allow extending the operating regimes of industrial engines towards more efficient fuel-lean conditions. Thus, our study opens up new avenues to develop and design more environmental friendly combustors.

## Methods

### Experimental setup

**Figure 7:**
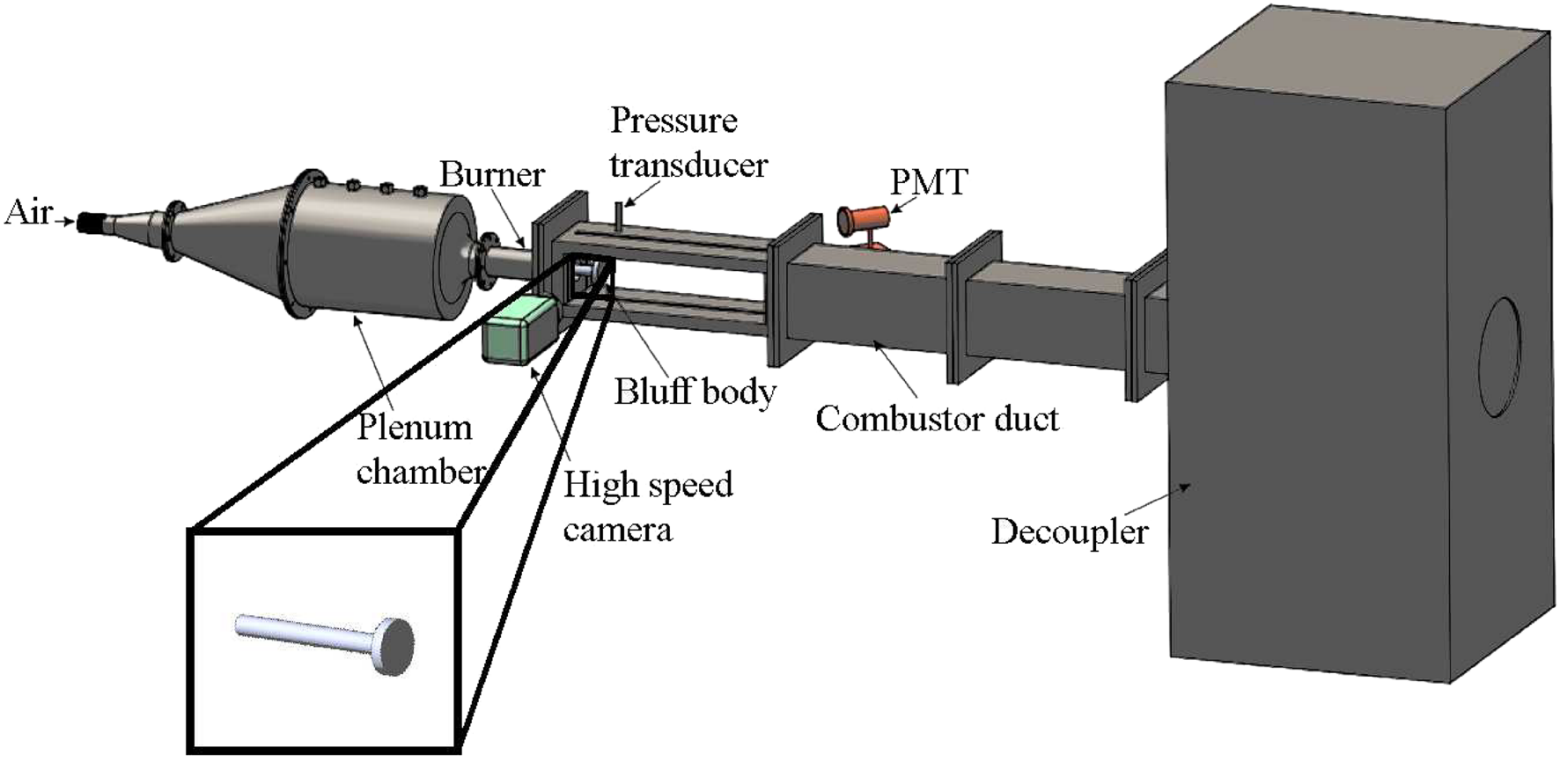
Schematic of the bluff-body stabilized turbulent thermoacoustic system. The experimental setup is a turbulent combustor that consists mainly of a plenum chamber, a burner and a combustion chamber. We utilize a piezoelectric pressure transducer, a photomultiplier tube and a high-speed camera to record data simultaneously. The inset shows a zoomed version of the bluff-body.

The components of the experimental setup and the data acquisitions systems are shown in Fig. 7. The circular bluff body that stabilizes the flame has a diameter of 40 mm and is mounted on a shaft with a diameter 16 mm. The combustion chamber is operated at atmospheric pressure and at turbulent conditions (Reynolds number *Re* > 18,000).

We acquire the acoustic pressure fluctuations $p^{\prime }$ using a piezoelectric pressure transducer (PCB103B02, uncertainty ± 0.15 Pa). In order to record the maximum amplitude of the standing wave, the pressure sensor is mounted at the antinode of the acoustic oscillations (25 mm distance from the backward-facing step of the combustor). Further, the piezoelectric transducer is mounted on a pressure port (T-joint) that is flush mounted on the combustor wall. To protect the transducer from excess heating from the combustor, a teflon adapter was also used. Further, one shoulder of the T-joint was also provided with a semi-infinite tube 10 m in length to prevent acoustic resonance within the ports thereby minimizing the frequency response of the system. The voltage signals from the piezoelectric transducer were recorded utilizing a 16-bit A-D conversion card (NI-643) with an input voltage range of 5 V and a resolution of 0.15 mV.

We acquire the global heat release rate $\dot{Q}$ by employing a photomultiplier tube (PMT, Hamamatsu H10722-01) outfitted with a CH* filter (435 nm ± 12 nm FWHM) in front of it. We also obtain spatial chemiluminescence intensities that are representative of the local heat release rate ($\dot{q}({\mathbf{x}} ,t)$) by utilizing a high-speed camera (of spatial resolution 800 × 600 pixels). Other studies have used chemiluminescence intensities as indicators of heat release rate for partially premixed flames^70,71^.

Terming the chemiluminescence intensities as a representative of heat release rate for partially premixed flames is questionable. However, the analysis of fluctuations that we perform depend on relative changes rather than absolute values. The chemiluminescence intensities obtained by the camera suffice as a measure of the spatiotemporal dynamics. Thus, whether the chemiluminescence intensities describe the absolute values of heat release rate is of lesser importance for our analysis. Further, even though the camera is not intensified, the measurement noise in the system is not large enough to significantly affect our analysis. Additionally, for our experimental data, the integral chemiluminescence intensities (obtained from images) have high correlation with the global heat release rate (obtained from PMT) at various equivalence ratios.

We utilize air as the oxidizer and liquefied petroleum gas (LPG: butane 60% and propane 40%) as the fuel for combustion. We vary the air-fuel mixture ratio by varying the mass flow rate of air $\dot{m}_{air}$ to change the state of the system from *S*_*CN*_ to *S*_*TI*_. The mass flow rates of air and fuel are controlled utilizing PID based mass flow controllers (MFC) having a rise time of 100 ms. In each experiment, we maintain the $\dot{m}_{fuel}$ constant and is input through the MFC software just prior to ignition of the flame. To control air mass flow rate, we utilize a data acquisition system (NI DAQ) to send inputs to the MFC in the form of voltage units. The minimum voltage of 0 corresponds to 0 SLPM and 5 V corresponding to 2000 SLPM. After ignition of the flame, the air mass flow rate is increased linearly at 3 SLPM/s utilizing a ramp signal given as input to the MFC controller from an NI DAQ.

We acquire the pressure and global heat release rate fluctuations at a sampling rate of 10 kHz, while we record the images of local heat release rate at a sampling rate of 500 frames per second. We acquire all the data simultaneously. Due to memory constraints, the sampling rate of of the camera for the experiments reported in this study is low considering that the dominant frequency is approximately 134 Hz. However, we have recorded chemiluminescence images at 2000 Hz for other quasi-steady experiments and we observe similar spatiotemporal patterns in the local heat release rate that we report in this study. The experimental configuration and instrumentation that we use in this work are similar to our previous study^36^.

### Variance of fluctuations of local heat release rate

We calculate the variance of fluctuations *σ*^2^ of $\dot{q}({\mathbf{x}} ,t)$ at each pixel for a time period of *w* = 3.5 s wherein the fluctuations are calculated based on a moving average of *w*_*s*_ = 0.04 s. *d* refers to the time instant at which the calculation is performed. *RP* is the reference point that is arbitrarily chosen to estimate the growth of *σ*^2^.

1 $$\begin{aligned} \sigma ^2(\dot{q}, d, w, w_s) = \sum _{k=1}^{w}\frac{\left[ \dot{q}(RP-d-k)-\sum _{i=1}^{w_s}\frac{\dot{q}(RP-d-k-i)}{w_s}\right] ^2}{w} \end{aligned}$$

For example, assuming *RP* is at 25 s, to calculate *σ*^2^ at 20 s (*d*) before the *RP*, we utilize the fluctuations from *t* = 1.5 s to *t* = 5 s. Next, we calculate *σ*^2^ at 10 s (*d*) before the *RP*, we utilize the fluctuations from *t* = 11.5 s to *t* = 15 s. Subsequently, we obtain the growth of *σ*^2^ from *t* = 20 s to *t* = 10 s before *RP*.

## Supplementary Information

Supplementary Information.

## Data Availability

All the data presented in this paper are available from the corresponding author on reasonable request.
