# Supplementary material for: Preventing a global transition to thermoacoustic instability by targeting local dynamics
Source: Sci Rep. 2022 Jun 3;12:9305. doi: 10.1038/s41598-022-12951-6 (PMC9166721; doi:10.1038/s41598-022-12951-6)
Supplement: Supplementary file 1 — Supplementary Information. [file 41598_2022_12951_MOESM1_ESM.pdf]

Preventing a global transition to thermoacoustic instability by targeting local dynamics

Nitin Babu George[1,2,*], **Manikandan Raghunathan**[3], **Vishnu R Unni**[4], **R. I. Sujith**[3], **Jürgen Kurths**[1,2], **and Elena Surovyatkina**[1,5]

[1]Potsdam Institute for Climate Impact Research, Potsdam, Germany
[2]Department of Physics, Humboldt University of Berlin, Berlin, Germany
[3]Department of Aerospace Engineering, Indian Institute of Technology, Madras, India
[4]Department of Mechanical and Aerospace Engineering, Indian Institute of Technology, Hyderabad, India
[5]Space Research Institute of Russian Academy of Sciences, Moscow, Russia
[*]george@pik-potsdam.de

Supplementary Information

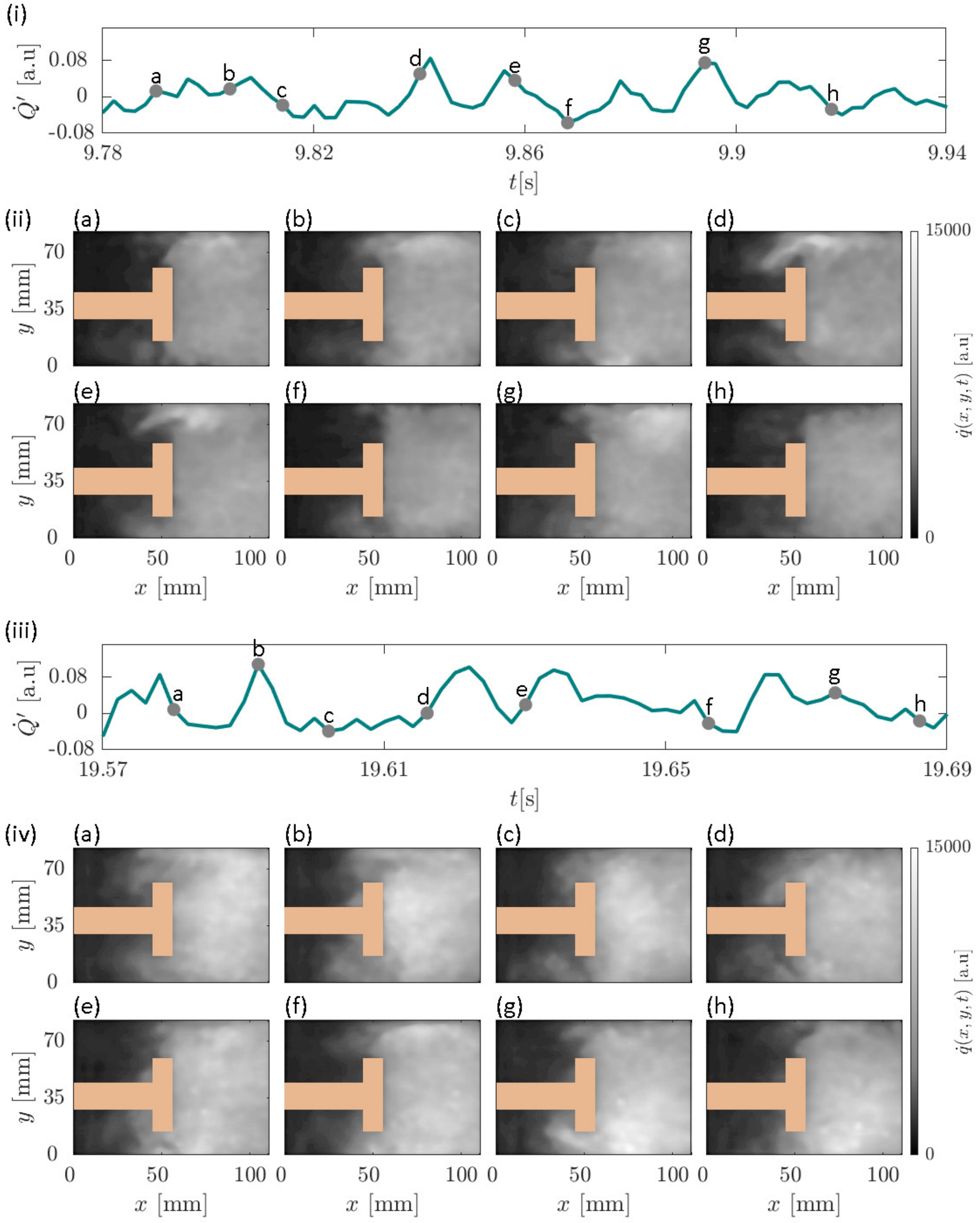

Figure S1. Fluctuations of the global heat release rate $\dot{Q}'$ and the corresponding images of instantaneous local heat release rate. i) Time series of $\dot{Q}'$ near t = 10 s. ii) Snapshots of the chemiluminescence images (a - h) corresponding to the time stamps shown in i). iii) Time series of $\dot{Q}'$ near t = 20 s. iv) Snapshots of the chemiluminescence images (a - h) corresponding to the time stamps shown in ii).

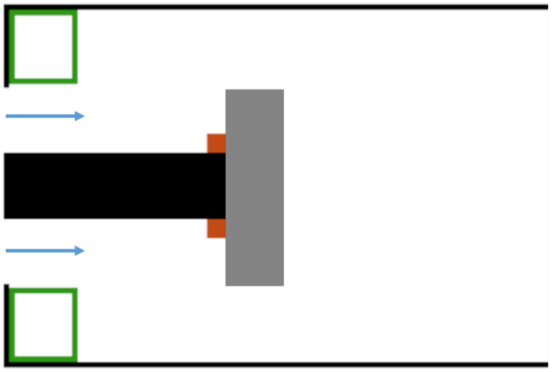

Figure S2. Schematic of the entry section of the turbulent combustor. The blue arrows show the direction of the bulk flow of the reactants into the rectangular duct. This combustor contains a bluff body to stabilize the flame. The bluff body, denoted by the grey surface is supported by a shaft, denoted by a black color. Fluid flows around the bluff body. This results in stagnation points illustrated using orange color at the corner between the shaft and the bluff body. Inlet conditions also result in a recirculation zone at the corner of the rectangular duct. Green squares represent the location of these recirculation zones. The turbulent combustor that we utilize is a backward-facing step combustor. The rectangular duct of the combustor has a cross section of 90×90 mm^2 and a total length of 1100 mm. As a result of the backward facing step at the inlet, a recirculation zones form at the corners. The fuel, which is a mixture of propane and butane is injected and mixes with air 120 mm upstream of the bluff body. As a result, we obtain a partially premixed flame.

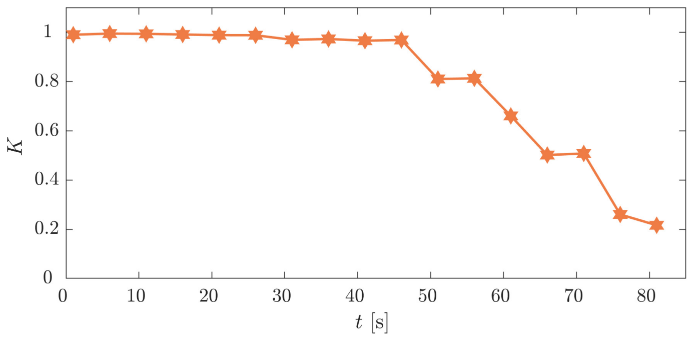

Figure S3. The 0-1 test for Chaos. Variation of K calculated from the acoustic pressure signal of the baseline bluff body with respect to t. K shifts from high value to low value as the system dynamics changes from combustion noise to thermoacoustic instability.

The 0-1 test distinguishes chaotic signals from regular time series. Values of growth rate of the mean square displacement (K) close to 1 indicate that the signal is chaotic while a K value close to 0 means that the signal is regular[1]. Figure S3 shows the variation of the median of K as a function of the mass flow rate of air. Close to $t = 0$, which is during combustion noise, K is close to 1 while at large t, K decreases to values near 0. This shift in K shows the loss of chaotic dynamics, when the system dynamics changed from combustion noise to thermoacoustic instability, similar to the observations in the study by Nair et al.[2]. For additional evidence, we perform an analysis of the maximum Lyapunov exponent[3] using methods provided by Rosenstein et al.[4] and Eckmann et al.[5]. A positive maximal Lyapunov exponent indicates chaotic dynamics of the system. The application of both methods for the time series of acoustic pressure fluctuations from t = 0 to 5 s shows that the maximum Lyapunov exponent is 0.005, suggesting that the signal is indeed chaotic. For detailed analysis to test for determinism in the acoustic pressure time series, we refer the reader to Tony et al.[6]. Utilizing surrogate methods as well as other discriminating measures such as permutation spectrum, correlation dimension, they showed that the time series of acoustic pressure during the state of combustion noise exhibit high-dimensional chaos.

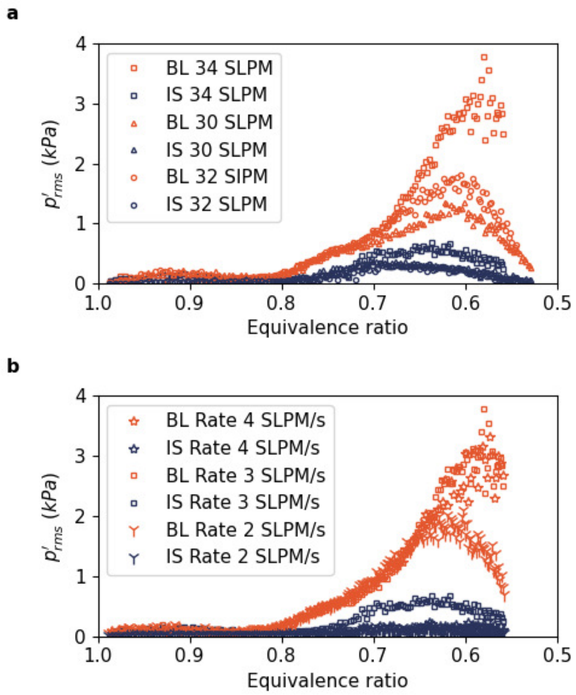

Figure S4. p'_{rms} for BL and IS bluff bodies at different operating conditions. **a** p'_{rms} for different $\dot{m}_{fuel}$: 30 SLPM, 32 SLPM and 34 SLPM at the same increase in air flow rate of 3 SLPM/s. For various $\dot{m}_{fuel}$ that we have utilized, the suppression of the onset of thermoacoustic instability occurs for the IS bluff body. As $\dot{m}_{fuel}$ increases, there is greater suppression of the pressure oscillations if we compare the maximum amplitudes of pressure oscillations obtained for each experiment. **b** p'_{rms} for different rate of increase of $\dot{m}_{air}$ 2 SLPM/s, 3 SLPM/s and 4 SLPM/s at the same $\dot{m}_{fuel}$ 34 SLPM. In these cases as well, we observe suppression for all the experiments with the IS bluff body.

References

1. Gottwald, G. A. & Melbourne, I. The 0-1 test for chaos: A review. *Chaos detection predictability* 221–247 (2016).
2. Nair, V., Thampi, G., Karuppusamy, S., Gopalan, S. & Sujith, R. I. Loss of chaos in combustion noise as a precursor of impending combustion instability. *Int. J. Spray Combust. Dyn.* **5**, 273–290 (2013).
3. Lyapunov, A. M. The general problem of the stability of motion. *Int. journal control* **55**, 531–534 (1992).
4. Rosenstein, M. T., Collins, J. J. & De Luca, C. J. A practical method for calculating largest lyapunov exponents from small data sets. *Phys. D: Nonlinear Phenom.* **65**, 117–134 (1993).
5. Eckmann, J.-P., Kamphorst, S. O., Ruelle, D. & Ciliberto, S. Liapunov exponents from time series. *Phys. Rev. A* **34**, 4971 (1986).
6. Tony, J., Gopalakrishnan, E., Sreelekha, E. & Sujith, R. Detecting deterministic nature of pressure measurements from a turbulent combustor. *Phys. Rev. E* **92**, 062902 (2015).
